# Midterm outcomes of 455 patients receiving the AFX2 endovascular graft for the treatment of abdominal aortic aneurysm: A retrospective multi-center analysis

**DOI:** 10.1371/journal.pone.0261623

**Published:** 2021-12-31

**Authors:** Raymond Vetsch, Harvey E. Garrett, Christopher L. Stout, Alan R. Wladis, Matt Thompson, Joseph V. Lombardi

**Affiliations:** 1 Freeman Heart & Vascular Institute, Joplin, Missouri, United States of America; 2 Department of Vascular Surgery, University of Tennessee Health Science Center, Memphis, Tennessee, United States of America; 3 Ozark Regional Vein and Artery Center, Rogers, Arkansas, United States of America; 4 Advent Health Medical Group, Orlando, Florida, United States of America; 5 Department of Vascular Surgery, Heart and Vascular Institute, Cleveland Clinic Foundation, Cleveland, Ohio, United States of America; 6 Cooper University Healthcare, Camden, New Jersey, United States of America; NIHR Leicester Biomedical Research Centre, UNITED KINGDOM

## Abstract

Since being introduced into clinical practice the AFX family of endografts has undergone labelling updates, design and manufacturing changes to address a Type III failure mode. The published literature on the performance of the current endograft–AFX2 –is limited to small series with limited follow up. The present study reports the largest series of patients implanted with AFX2 for the treatment of abdominal aortic aneurysms. The study was a retrospective, 5 center study of patients receiving an AFX2 endograft from January 2016 until Dec 2020. Electronic case report forms were provided to four of the centers, with one additional site providing relevant outcomes in an independent dataset. Relevant outcomes were reported via Kaplan-Meier analysis and included all-cause mortality, aneurysm-related mortality, post EVAR aortic rupture, open conversion, device related reinterventions and endoleaks. Among a cohort of 460 patients, 405 underwent elective repair of an AAA, 50 were treated for a ruptured AAA, and 5 were aorto-iliac occlusive disease cases. For the elective cohort (mean age 73.7y, 77% male, mean AAA diameter 5.4cm), the peri-operative mortality was 1.7%. Freedom from aneurysm-related mortality was 98.2% at 1,2,3 and 4 years post-operatively, there were no post-operative aortic ruptures, and 2 patients required open conversion. Freedom from Type Ia endoleaks was 99.4% at 1, 2, 3 and 4 years. Freedom from Type IIIa and Type IIIb endoleaks were 100% and 100% (year 1), 100% and 99.6% (year 2), 99.4% and 99.6% (year 3), 99.4% and 99.6% (year 4) respectively. Freedom from all device-related reintervention (including Type II endoleaks) at 4 y was 86.8%. The AFX2 endograft appears to perform to a satisfactory standard in terms of patient centric outcomes in mid-term follow up. The Type Ia and Type III endoleaks rates at 4y appear to be within acceptable limits. Further follow up studies are warranted.

## Introduction

Endovascular aneurysm repair (EVAR) is the dominant modality of abdominal aortic aneurysm (AAA) repair in the USA [1]. A successful EVAR procedure is partially predicated on achieving proximal fixation and aortic seal to effectively exclude the aneurysm from the circulation. The majority of endografts used for EVAR have a design that includes an active mechanism for supra-renal or infra-renal fixation in the proximal aorta [2]. In contrast to these proximally fixated grafts, the AFX2 endograft (Endologix, Irvine, CA, USA), uses anatomical fixation on the aortic bifurcation with a modular proximal cuff for aortic seal. The AFX2 system also uses a metallic endoskeleton with the graft fabric attached in several places but free to move over much of the endograft stent. In contrast, the proximally fixated endografts have an exoskeleton with firmly adherent graft material.

The AFX family of endografts was introduced into commercial practice in the USA in 2011 and the earliest version of this endograft (AFX Strata) had a failure mode of Type III endoleaks [3] which appeared to have a higher incidence than observed in proximally fixated grafts. Since commercialization the AFX endograft has undergone labelling updates, design and manufacturing changes [4], that have culminated in the currently commercialized AFX2 endograft.

The published literature on the performance of the AFX2 endograft is limited [5–9], but a recent publication by Chang et al. (2021) [10] which reported a single integrated healthcare system experience of AFX and AFX2, suggested that the AFX2 endograft was associated with a high rate of adverse clinical events during follow up. The present study was designed to evaluate the outcomes derived by the AFX2 endograft in a series of patients from multiple centers in the USA.

## Methods

### Study design

The present study was a retrospective, multicenter study of patients receiving an AFX endograft from January 2016 until Dec 2020. From a pragmatic perspective, due to the commercial profile, the vast majority of patients receiving an AFX endograft after February 2016, would have received the AFX2 endograft as opposed to a previous iteration of the AFX family of endografts. The study was performed in 5 USA centers. Advarra central IRB approval with waiver of patient consent was obtained. No patient identifiers are included in data collection as identified in the protocol.

### Study population

The study population included patients implanted with an AFX endograft for the treatment of an AAA within the study dates at the participating U.S. centers. Patients with an elective procedure for an unruptured AAA were reported and analyzed separately from those patients with a ruptured AAA. Patients who had a revisional procedure or had an AFX endograft as part of a procedure for aorto-iliac occlusive disease were excluded. Patients were identified and cross referenced from institutional databases and commercial sales data. Treatment algorithms, including device sizing, technical implantation procedure, follow up imaging and clinical follow up protocol were at the discretion of the implanting site and were reflective of individual institutional protocols. All outcome measures were site reported, collected retrospectively and entered into an electronic data management system for analysis. All sites adhered to established guidelines with regard to post-operative imaging and surveillance [11, 12]. No detailed analysis of pre-operative aneurysm morphology was available in the study and there was no information as to conformance with the anatomical indications for use.

### Reported outcomes

Relevant outcomes analyzed included all-cause mortality, aneurysm-related mortality, post EVAR aortic rupture, open conversion, device-related reinterventions (defined as all interventions related to the device and/or abdominal aortic aneurysm with the exception of Type II endoleaks), graft occlusion, sac enlargement and endoleak as per established reporting standards [13]. With the exception of one single center, Type I and type III endoleaks were classified into their a and b sub-classifications as per the same reporting standards.

### Statistical analysis

Continuous variables are presented as mean values with standard deviation. Freedom from adverse events (all-cause mortality, aneurysm-related mortality, open conversion, device related reinterventions, graft occlusion, sac enlargement and endoleak) are reported using Kaplan-Meier survival analysis, with numbers of patients at risk at each follow up period presented. Patients were censored at their last follow-up. All analyses were performed in SAS version 9.4. Copyright © 2021 SAS Institute Inc. SAS and all other SAS Institute Inc. product or service names are registered trademarks or trademarks of SAS Institute Inc., Cary, NC, USA.

## Results

### Elective aneurysm repair

There were 405 patients who underwent elective repair of an intact AAA (mean AAA diameter 54mm ± 10. The mean age of the patients was 73.7 years ± 8.8 (mean ± SD) and 77% were male. Three hundred and fifty-two (86.9%) patients had recorded clinical or imaging follow up after the peri-operative period. The mean follow-up of the elective cohort was 1.7 years ± 1.3 (mean ± SD). Expressed on an annual basis, 246, 157, and 71 patients had follow-ups at or beyond 1, 2, and 3 years respectively. There were 7 peri-operative death within the elective cohort (1.7%) and 6 in the emergent group (12%). Within the elective cohort these deaths resulted from vascular complications (2 intestinal ischaemia, 2 vascular dissection and/or hemorrhagic shock, multiorgan failure) or unknown causes [2]. The most common peri-operative adverse events included Type Ia endoleak (n = 6, 1.5%), femoral artery pseudoaneurysm (n = 4, 1%), limb thromboses (n = 3, 0.7%) and renal artery stenosis/occlusion (n = 2, 0.5%).

### All-cause mortality and aneurysm-related mortality

**Fig 1:**
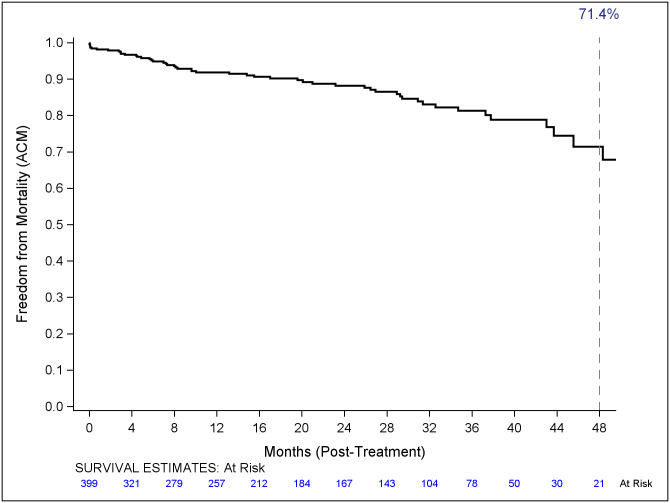
Freedom from all cause mortality. Kaplan-Meier curve illustrating freedom from all-cause mortality to 48 months. Numbers of patients at risk are presented on the x axis.

**Fig 2:**
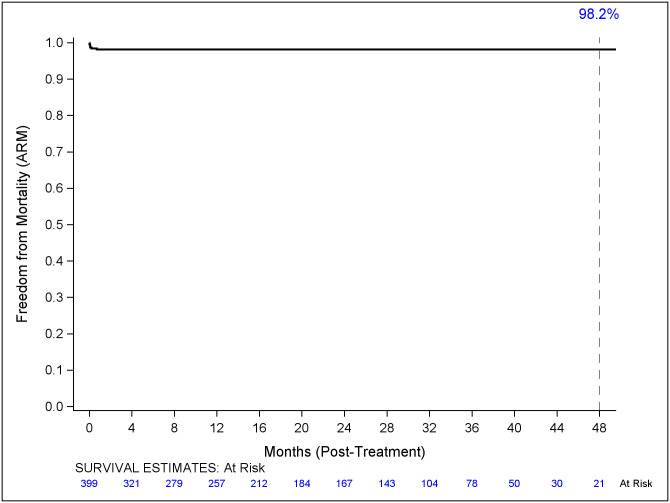
Freedom from aneurysm related mortality. Kaplan-Meier curve illustrating freedom from aneurysm-related mortality to 48 months. Numbers of patients at risk are presented on the x axis.

Freedom from all-cause mortality and aneurysm-related mortality is illustrated in Figs 1 and 2. Freedom from all-cause mortality at 1,2,3 and 4 years was 91.8%, 88.2%, 81.3% and 71.4% respectively. Freedom from aneurysm-related mortality was 98.2% at 1,2,3 and 4 years post-operatively.

### Open conversion and aortic rupture

In the patients undergoing elective repair of an unruptured AAA, there were no aortic ruptures in follow up out to 4 years. Freedom from open conversion was 98.8% at 2, 3 and 4 years. The two conversions seen were for sac expansion secondary to a Type II endoleak and graft infection.

### Post-operative endoleaks and device related reintervention

Freedom from Type Ia endoleaks was 99.4% at 1, 2, 3 and 4 years. Freedom from Type Ib endoleaks at 1,2,3 and 4 years follow-up was 100%, 99.6%, 99.6% and 94.3% respectively. Overall freedom from all Type I endoleaks was 99.5%, 99.1%, 99.1% and 93.8% at 1, 2, 3 and 4 years.

Freedom from Type IIIa and Type IIIb endoleaks at the same time points were 100% and 100% (year 1), 100% and 99.6% (year 2), 99.4% and 99.6% (year 3), 99.4% and 99.6% (year 4) respectively. The freedom from Type III endoleaks overall was 100%, 99.6%, 98.9% and 98.9% at 1,2,3 and 4 years. The single site that did not stratify Type I and Type III endoleaks was removed from the at-risk population for the purposes of the separate Type Ia and Type Ib Kaplan-Meier analyses, but not removed from the Type IIIa and Type IIIb analyses as this site reported 0 type IIIs.

Freedom from Type II endoleaks was 97.7% at year 1, 94.3% at year 2, 91.8% at year 3 and 87.0% at year 4. There were no Type IV endoleaks or endoleaks of unknown etiology in the study.

**Fig 3:**
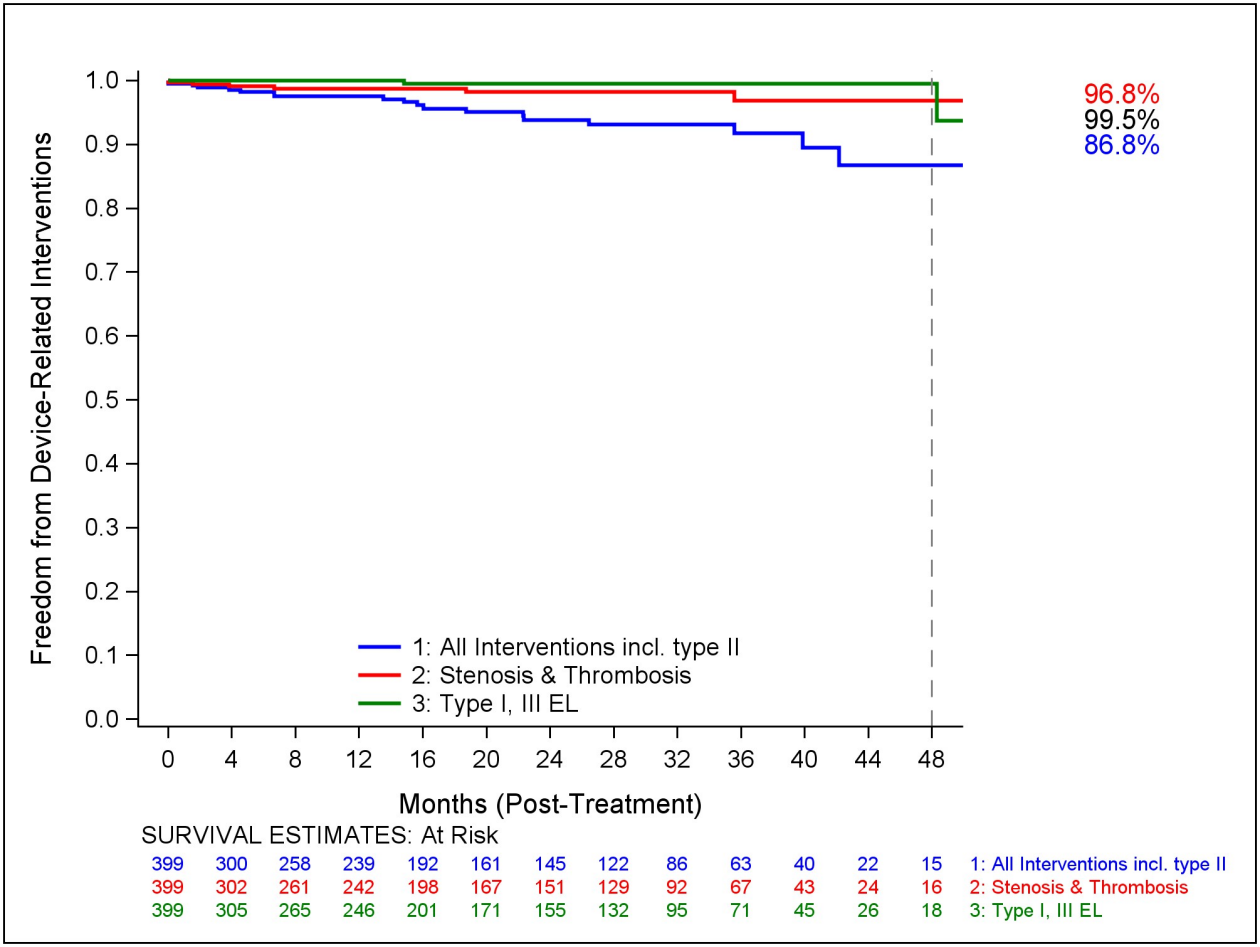
Freedom from AAA-related interventions.

**Fig 4:**
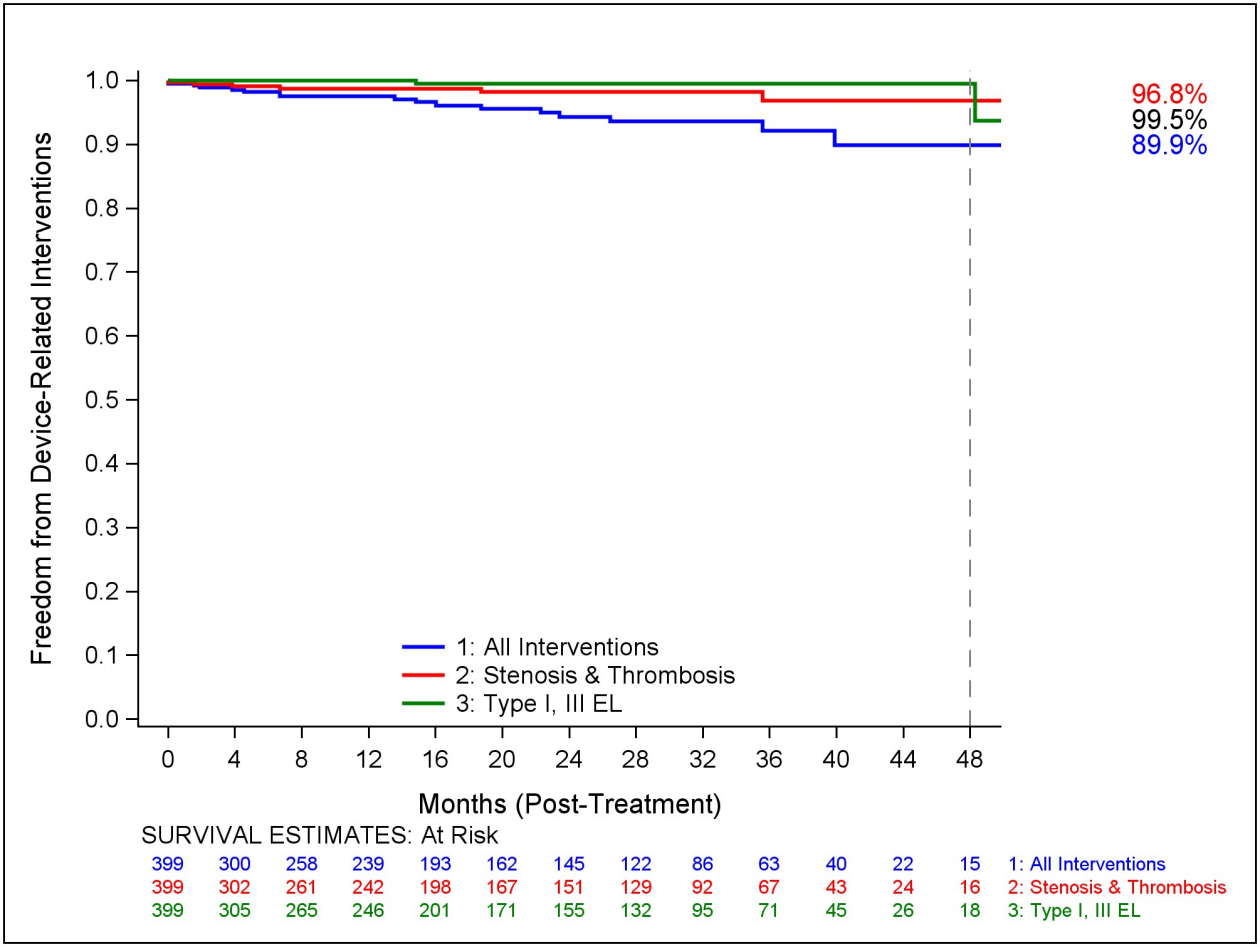
Freedom from device-related interventions. Kaplan-Meier curve illustrating freedom from all device related reintervention including (Fig 3) and excluding interventions (Fig 4) for Type II endoleaks. Freedom from reintervention for endoleaks Types I/III and for graft stenosis / occlusion have been defined separately. Numbers of patients at risk are presented on the x axis.

Freedom from all device-related reintervention including and excluding interventions for Type II endoleaks is illustrated in Figs 3 and 4 respectively. Freedom from reintervention for endoleaks Types I/III and for graft stenosis / occlusion have been defined separately. The most common complications requiring reintervention were sac enlargement (n = 3, 0.7%), graft stenosis/occlusion (n = 10, 2.5%), Type II endoleaks (n = 6, 1.5%), and Type I/III endoleaks (n = 2, 0.5%). The most common reinterventions identified were embolization (n = 7, 1.7%), PTA/stent (n = 9, 2.2%), iliac extension (n = 7, 1.7%), thrombectomy/thrombolysis (n = 3, 0.7%), and aortic extension cuff (n = 2, 0.5%).

### Repair of ruptured abdominal aortic aneurysm

Fifty patients underwent repair of ruptured AAA as an emergent procedure during the study period. The mean age of the patients was **72.7 +/- 10.5 (mean +/- SD)** with **26%** being male. The mean aneurysm diameter was 63mm (**SD: 20 mm**). The peri-operative mortality was 10% (5 patients). The mean follow-up of the patients with a ruptured AAA was 12 months, and insufficient patients were available after this 12 month time period to give any longer term information on endoleaks. The 12 month aneurysm-related outcomes are tabulated in Table 1 with the corresponding 12 month rates from the elective cohort. As illustrated, all outcomes were worse in the ruptured cohort.

**Table 1:** Freedom from aneurysm related outcomes.

Freedom from	Emergent Treatment for Ruptured AAA (n = 50)	Elective Treatment for Intact AAA (n = 405)
All-cause mortality	80.1%	91.8%
Aneurysm related mortality	89.8%	98.2%
Open conversion	100%	100%
Aortic rupture	100%	100%
Endoleak Type I	95%	99.5%
Endoleak Type III	100%	100%
Device related secondary intervention	97.4%	97.5%

Table reporting the aneurysm related outcomes at 1 year for patients treated with the AFX2 graft for ruptured abdominal aortic aneurysms in the present study, compared to outcomes at the same time points for patients treated electively for intact AAA.

## Discussion

The present study reports the largest series of patients receiving the AFX2 endograft for the treatment of AAA. The mid-term outcomes of these patients appear satisfactory with low rates of aortic related death, post EVAR aortic rupture and device related reintervention. Additionally, the rate of Type I and Type III endoleak was observed to be below or consistent with rates derived from previous literature as related to other endograft designs [14–16].

The AFX family of endografts was introduced into commercial practice in 2011 and succeeded the Powerlink system which had a reasonable body of clinical data and a low rate of Type III endoleaks [17–19]. Following introduction of the initial AFX graft, there were reports of Type III endoleaks and these were confirmed through internal investigation and in subsequent publications [3, 20]. In order to mitigate the Type III failure mode, the AFX endograft system underwent labelling, design and manufacturing changes. These changes and the nomenclature associated with the version of the AFX family are illustrated in Table 2, and culminated in the AFX2 endograft, which is the only currently available product.

**Table 2:** AFX endografts.

AFX Version	Labelling update, design, or manufacturing change	Date of labelling update or change
AFX Strata	Prior to any corrective actions	July 2011
AFX Strata	Labelling update—overlap recommendations to mitigate Type IIIA endoleak	May 2013
AFX Duraply	Manufacturing change—Duraply ePTFE	July 2014
	Labelling update—Type IIIb endoleaks	
AFX Duraply	Labelling update—oversizing and patient selection recommendations	Sept 2015
AFX2	Labelling update—sizing algorithm	Feb 2016
	Design change—increased PTFE thickness	
	Design change—delivery catheter change to protect bifurcation	

Table documenting the versions of the AFX family of endografts, the labelling updates, design and manufacturing changes with associated dates.

The AFX family of endografts has a unibody design, which allows some unique applications in patients with a narrow distal aorta [7, 21] and in patients in whom preservation of the native aortic bifurcation is required for future treatment of peripheral vascular disease [22].

With recent publications raising concerns about the durability of EVAR in general [23], defining outcomes through the use of several data sets becomes important. In general, the outcomes of EVAR for any particular device should ideally be evaluated using multiple datasets, some of which would include an assessment of comparative graft performance. It appears relevant that different endografts will have differing design intentions and consequently a spectrum of failure modes that is unique to that endograft. For this significant reason it is important that endograft outcomes are evaluated using a holistic assessment of graft function with single failure modes being weighed appropriately.

Given that the early versions of AFX had a higher than anticipated Type III endoleaks rate and that Type III endoleaks occur relatively late after initial implantation, the question arises as to whether the changes to the AFX platform have sufficiently mitigated the failure mode in the currently available AFX2 endograft. In a recent publication, Chang et al. (2021) [10] suggested that AFX2 had a continued intrinsic issue as they reported that the 2-year incidence of Type III endoleak was 14.1% in a cohort of 33 patients analyzed retrospectively with 14 followed out to 2 years. The equivalent 2-year rates for all Type III endoleaks, Type IIIa endoleaks and Type IIIb endoleaks from the present study were 0.4%, 0% and 0.4% respectively, with the 4-year rates for the same categories being 1.1%, 0.6% and 0.4%. Clearly, the incidence of Type III endoleak observed in the present study as compared to the observations from Chang et al. (2021) are at least an order of magnitude different.

One of the criticisms of reports detailing the results of elective EVAR is the focus on endoleaks without a description of the consequences of such complications [24]. From a patients perspective, of more relevance than endoleak, are the more injurious events of reintervention, aortic rupture and aortic related mortality [24]. The data from Chang et al. (2021) demonstrated that the 2-year incidence of device related reintervention, aortic rupture and aortic related mortality was 16.2%, 7.3% and 6.1% respectively. The corresponding rates from the present study were 5.7%, 0% and 1.8%.

Clearly, there is a large discrepancy in the outcomes reported by Chang et al. (2021) and the current study. Part of the explanation for the variation in incident rates is the low number of patients in the Chang et al. (2021) study as well as other limitations addressed in the publication such as a lack of endoleak sub-classification, the possibility that endoleaks were mis-classified, the indication for surgery, anatomy and surgical technique. Given the magnitude of difference in the outcomes reported in the present study in comparison to the data from a single hospital system, it seems reasonable to attempt to define which of the experiences reflects real world practice with reference to other available datasets.

In this regard, the LEOPARD trial [25] is an interesting comparator. The Endologix sponsored LEOPARD trial is the only trial pertaining to EVAR, that uses the methodology of a randomized controlled trial to evaluate comparative graft performance. The LEOPARD trial randomized patients between AFX Duraply/AFX2 and a commercially available comparator endograft (Medtronic, Dublin, Ireland; WL Gore and Associates, Flagstaff, AZ; Cook Group, Bloomington, IN) that was pre-specified by each investigator at the commencement of the study. The trial was a real world study, with the investigators determining the suitability for EVAR. The primary end point was freedom from aneurysm related complications at one year.

The three year data from the LEOPARD trial are publicly available [4] and the main outcome measures for the AFX platform from both the LEOPARD study and the present trial are tabulated in Table 3. There is a high degree of concordance between the data from the present study and the incident rates reported in the LEOPARD trial. The LEOPARD study reflects Level 1 clinical evidence [26] and was a real-world study with 235 patients implanted with AFX Duraply / AFX2, by 105 investigators at 56 sites. The analysis of LEOPARD data has historically been based on site-reported data, but an independent core laboratory for image analysis is providing additional data, and adverse events were independently adjudicated. The quality of evidence from the LEOPARD study, and the similarity to the outcomes reported in the present study give assurance that the rates of adverse outcomes reported in the present study are reflective of those observed with the AFX2 endograft in multiple centers across the USA, although further studies in this regard would be important [27].

**Table 3:** Freedom from adverse events.

Freedom from	Present Study	LEOPARD Trial
All-cause mortality	81.3%	84.2%
Aneurysm related mortality	98.2%	98.2%
Open conversion	98.8%	100%
Aortic rupture	100%	99.5%
Endoleak Type I	99.1%	97.1%
Endoleak Type III	98.9%	99.5%
Device related secondary intervention	92.2%	89.8%

Table illustrating freedom from adverse events at 3 year derived from the LEOPARD study and the present study.

The present study gives a limited amount of information regarding the use of AFX2 in the treatment of ruptured AAA. The peri-operative mortality rate is low at 10% but this likely reflects patient selection in real-world practice where mortality rates are often much lower than in the randomized controlled trials [28–30]. The information on post-operative aneurysm-related outcomes and endoleaks is confined to a short follow up period. However, all outcomes at one year are worse than in the elective cohort, which is consistent with previous reports [31].

The limitations of this study are typical of other device studies and the inherent nature of a retrospective study. One major limitation is that the study was funded by the medical device manufacturer responsible for the AFX2 product and the company participated in study design, data acquisition, data analysis and writing of the manuscript.

Due to the retrospective nature of this multi-center study, the patient demographics and the aortic anatomy were not well characterized. As such, this multi-center study was not able to define access or aortoiliac anatomy well in addition to patient demographics. The lack of information with regard to pre-operative aortic morphology, made it impossible to attribute any of the outcomes to a particular aortic risk factor or non-conformance to the anatomic indications for use. Additionally, there was a lack of risk factor classification, which does not allow for the patient population to be clearly defined in terms of co-morbidities. However, the all-cause mortality observed in the present study is similar to that reported in populations of patients with abdominal aneurysms [32] and so it seems likely that the present study involved a patient cohort with typical risk factors.

Given the study design and the retrospective nature of data acquisition, all outcomes were site reported and were not independently adjudicated. Similarly, a core laboratory was not used to verify imaging findings. The lack of independent data adjudication and study audit must be considered a limitation of the present study. As is typical with real-world practice, there will be a loss to follow up with patients generally having poor compliance with surveillance regimes [33, 34]. It remains possible that some of the non-compliant patients may have presented to hospitals outside of the study centers and that their complications remain unidentified in the present study. This issue is similar to that which is seen in other device studies.

Finally, there is a question as to how generalizable these results are to surgical practice in the USA and other countries. All of the institutions that contributed to the study would be considered high volume aortic centers and the reported outcomes would reflect this [35]. Similarly, the results may not be generalizable to global practice as there are significant differences in patient selection and reported outcomes between geographies [36].

## Conclusion

In the present study the AFX2 endograft performs to a satisfactory standard in terms of patient centric outcomes in mid-term follow up. The Type Ia and type III endoleaks rates at 4 years are consistent with prior studies and within acceptable limits of and comparable to other endografts on the market. However, due to the durability concerns with EVAR, we recommend prospective studies and continued retrospective long term follow-up studies.

## Supporting information

S1 Dataset (ZIP)
